# Montelukast and Telmisartan as Inhibitors of SARS-CoV-2 Omicron Variant

**DOI:** 10.3390/pharmaceutics15071891

**Published:** 2023-07-05

**Authors:** Nirmitee Mulgaonkar, Haoqi Wang, Junrui Zhang, Christopher M. Roundy, Wendy Tang, Sankar Prasad Chaki, Alex Pauvolid-Corrêa, Gabriel L. Hamer, Sandun Fernando

**Affiliations:** 1 Biological and Agricultural Engineering Department, Texas A&M University, College Station, TX 77843, USA; 2 Department of Entomology, Texas A&M University, College Station, TX 77843, USA; 3 Texas A&M Global Health Research Complex, Division of Research, Texas A&M University, College Station, TX 77843, USA; 4 Department of Veterinary Integrative Biosciences, Texas A&M University, College Station, TX 77843, USA

**Keywords:** SARS-CoV-2, COVID-19, montelukast, antiviral, drug repurposing

## Abstract

Earlier studies with montelukast (M) and telmisartan (T) have revealed their potential antiviral properties against SARS-CoV-2 wild-type (WT) but have not assessed their efficacy against emerging Variants of Concern (VOCs) such as Omicron. Our research fills this gap by investigating these drugs’ impact on VOCs, a topic that current scientific literature has largely overlooked. We employed computational methodologies, including molecular mechanics and machine learning tools, to identify drugs that could potentially disrupt the SARS-CoV-2 spike RBD-ACE2 protein interaction. This led to the identification of two FDA-approved small molecule drugs, M and T, conventionally used for treating asthma and hypertension, respectively. Our study presents an additional potential use for these drugs as antivirals. Our results show that both M and T can inhibit not only the WT SARS-CoV-2 but also, in the case of M, the Omicron variant, without reaching cytotoxic concentrations. This novel finding fills an existing gap in the literature and introduces the possibility of repurposing these drugs for SARS-CoV-2 VOCs, an essential step in responding to the evolving global pandemic.

## 1. Introduction

Over the past few years, several viral infectious diseases have occurred, causing a significant global threat to public health and the economy. Among these, the emergence and re-emergence of coronavirus-associated outbreaks triggering fatal respiratory diseases have created havoc worldwide [1]. Coronaviruses are a large group of enveloped, positive-sense single-stranded RNA viruses consisting of a genome of about 26–32 kilobases (kb), the second largest of reported RNA genomes [2,3]. Although coronaviruses are known to originally emerge from animal sources, seven coronavirus strains are known to infect humans. Of these, three strains, the 2003 severe acute respiratory syndrome coronavirus (SARS-CoV), the 2012 Middle East respiratory syndrome coronavirus (MERS-CoV) [4], and the 2019 SARS-CoV-2, cause severe symptoms and death. The ongoing coronavirus disease 2019 (COVID-19) pandemic is caused by a novel betacoronavirus, SARS-CoV-2, belonging to the *Sarbecovirus* subgenus, *Orthocoronavirinae* subfamily [5,6] has resulted in more than 6.8 million deaths globally, as of March 2023 [7]. To date, the wild-type strain has emerged into several variants that are circulating worldwide. Of these, the Omicron strain has been classified as a variant of concern (VOC) [8].

The SARS-CoV-2 genome (~30 kb) encodes four main structural proteins: spike (S), envelope (E), membrane (M), and nucleocapsid (N) that are responsible for virus attachment and virion assembly, a total of 16 nonstructural proteins (NSP1 to NSP16) that are primarily involved in RNA synthesis and processing, and accessory proteins [2]. The S protein mediates virus entry into the host cell. It consists of an N-terminal S1 subunit which is involved in virus attachment through its receptor-binding domain (RBD), while the C-terminal S2 subunit enables membrane fusion. Like SARS-CoV, SARS-CoV-2 also uses the ACE2 receptor for entry and the cellular serine protease TMPRSS2 for spike protein priming [9]. While the SARS-CoV-2 RBD binds to the ACE2 receptor with a nearly identical binding mode [10,11,12,13], its binding affinity is significantly higher as compared to SARS-CoV [12,13]. Furthermore, studies have shown that the omicron RBD binds to ACE2 with enhanced affinity and also elucidated the role of each of the omicron mutations in ACE2 binding [14].

**Figure 1:**
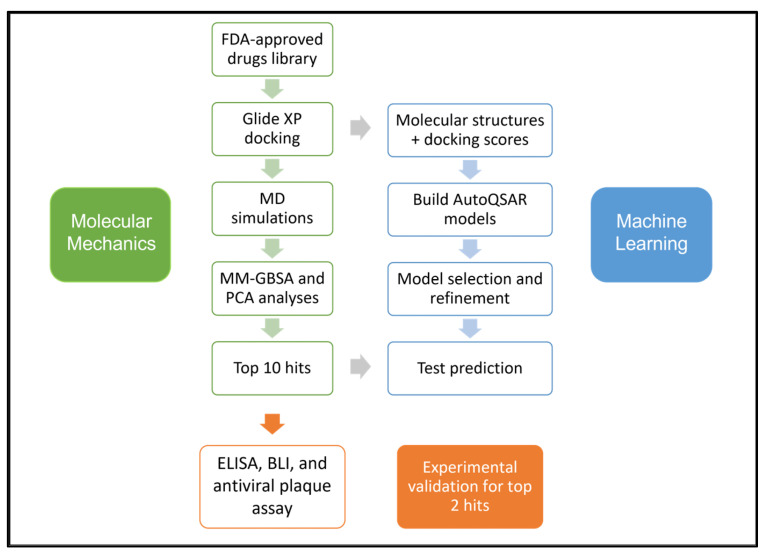
Schematic of the workflow used in this study.

Vaccines for the prevention of COVID-19 have been approved and are in use in many countries, while several other promising candidates are in trials [15,16]. Although the U.S. Food and Drug Administration (FDA) has approved remdesivir as the first treatment for COVID-19 [17], the scientific community is still debating over remdesivir’s efficacy [18]. Given the current situation of COVID-19 therapeutics, drug repurposing remains a viable strategy until specific antivirals against SARS-CoV-2 are developed. The S-protein, due to its significant role in virus-receptor interactions leading to virus entry, is a crucial target for therapeutic development. Here, we report a comprehensive computational workflow, as shown in Figure 1, for screening potential FDA-approved drugs targeting the SARS-CoV-2 spike RBD-ACE2 interaction interface that resulted in 10 hits. In vitro, experiments confirmed the efficacy of montelukast and telmisartan as potential SARS-CoV-2 inhibitors for WT and the Omicron variant. Previous studies have reported the efficacy of montelukast and telmisartan against WT [19,20,21], however, our study is the first to investigate their efficacy against VOCs, specifically the Omicron variant, presenting a significant addition to current knowledge.

## 2. Materials and Methods

### 2.1. Protein Structure

The three-dimensional structures of spike RBD (wild-type) (PDB:6VW1) [22] and Omicron (PDB:7U0N; chain E) [14] strain bound to the human ACE2 receptor were retrieved from the Protein Data Bank [23]. The protein structures were pre-processed, optimized, and minimized using Schrödinger’s Protein Preparation Wizard [24].

### 2.2. Molecular Docking

One hundred FDA-approved drugs from the high-throughput virtual screening process reported in the previous study [25] were re-docked against SARS-CoV-2 spike RBD in the presence of its cellular receptor ACE2 using Glide [26]. These molecules were subjected to an Extra Precision (XP) docking procedure. A grid box of size 32 × 32 × 32 Å^3^ was generated, centered at the residues where the RBD (Gln493-Gly496) was bound with ACE2. The best conformation for each drug was recorded from the resulting ten probable docked poses. The 100 molecules were re-ranked according to their Glide scores and drug interactions with the protein complex were analyzed using Schrödinger Maestro. Molecular dynamics simulations were performed to further evaluate the dynamic behavior and intermolecular interactions of protein-drug complexes for the top 10 ranked drugs. Docking results were also validated for the selected drugs against the RBD structures of SARS-CoV-2 wild-type, and Omicron strains using the above Glide XP protocol. To investigate the prophylactic potential of these molecules the docking protocol was also used for the ACE2 protein alone. The best-scored docking poses were rescored using the Prime/MM-GBSA platform for a better prediction of the binding affinity of ligands [27].

### 2.3. Molecular Dynamics (MD) Simulations

Most stable conformations of the ten selected drugs from the refined docking process were further subjected to 50 ns MD simulations which were performed using Schrödinger-Desmond [28]. The protein-drug complexes were prepared using Schrödinger’s Protein Preparation Wizard [24]. Structure refinement included capping the C and N termini of the protein, addition of missing hydrogens, hydrogen bond optimization, and minimization using the OPLS3e force field [29]. System Builder was used to build a system for each of the pre-processed protein-drug complexes and was solvated in an orthorhombic box using the Simple Point-Charge (SPC) solvent model with a buffer distance of 10 Å. The system charge was neutralized with sodium cations or chloride anions and a 0.1 M salt concentration was added. Desmond’s default relaxation protocol under the OPLS3e force field was used for performing the MD simulations. First, all heavy atoms in the system were minimized with restraints under 10 K, then further restrained by increasing the temperature to 300 K, and lastly relaxed under 300 K constant number of atoms, pressure, and temperature (NPT) ensemble to bring the system to its equilibrium condition. After relaxation, the systems were simulated under a 300 K NPT ensemble at 1.01325 bar pressure for 50 ns. Five hundred trajectory snapshots and associated energy terms were recorded at an interval of 100 ps. The post-simulation analyses, including complex root mean square deviation (RMSD), and root mean square fluctuation (RMSF) were done using Schrödinger’s Simulation Interaction Diagram (SID).

### 2.4. Principal Component Analysis (PCA)

To study the internal motion of the protein system from the overall dynamics of the simulation trajectory, a PCA of the pairwise distance of alpha carbon (Cα) atoms was performed. The changes in the conformation of a protein with respect to time are characterized by a vector space involving a large number of dimensions. PCA is a statistical technique applied to MD trajectories to reduce the dimensional space required to describe the essential motion of protein dynamics [30]. The topology and trajectory files were prepared by Visual Molecular Dynamics (VMD) [31] and the python package MDTraj [32] was used for analysis and visualization. The graphs were constructed with NumPy and matplotlib packages in python [33,34]. The interatomic distance of only Cα atoms was extracted from 500 snapshots of 50 ns MD trajectories to construct the covariance matrix. Each snapshot of the whole simulation was aligned to the initial frame and PCA was conducted to construct the eigenvectors. The first two principal components (PC1 and PC2) were predicted to represent conformational changes in the MD trajectory between the drug-protein system and the apo-protein system.

### 2.5. Binding Energy Calculations

The Molecular Mechanics-Generalized Born Surface Area (MM-GBSA) free energy of binding [35] was calculated over the MD trajectories of the last 25 ns of the simulation using the thermal_mmgbsa.py script from Schrödinger. Twenty-six frames (one every 10 frames) were selected from 250 frames for MM-GBSA calculation for each drug molecule. Prime MM-GBSA calculations use the VSGB 2.1 solvation model [36] under the OPLS3e force field. 

The binding free energy ΔG_bind_ was calculated using the equation below:

ΔG_bind_ = E_complex_ (minimized) − (E_ligand_ (minimized) + E_receptor_ (minimized)) (1)

The total ΔG_bind_ can be further decomposed into its contributing components:

ΔG_bind_ = E_coulomb_ + E_covalent_ + E_vdW_ + E_lipophilic_ + E_GB_solvation_ + E_corrections_ (2)

where, E_Corrections_ = E_Hbond_ + E_Pi-pi packing_ + E_Self-contact_ [36]. Although the MM-GBSA ΔG_bind_ values are reported in kcal/mol these values are different from the experimental binding free energy values. The MM-GBSA method accounts for molecular mechanics and solvation terms. The ΔG_bind_ values reported are based on end-point free energy calculations as a result of which the conformational entropy terms are omitted. This approximation causes the predicted ΔG_bind_ values to be more negative than the experimental values [37]. Since only relative ΔG_bind_ estimates are needed for virtual drug screening, the MM-GBSA method is considered a reliable method [38]. Two drugs, montelukast (M) and telmisartan (T), with the best MM-GBSA scores, were selected for experimental validation.

### 2.6. AutoQSAR Modeling

A simultaneous automated Quantitative Structure-Activity Relationship (QSAR) workflow was performed using the Schrödinger AutoQSAR platform [39], a machine learning tool. The molecular structures (~60 ligands) and binding affinity results from Glide docking were used as the input dataset. For model assessment, the input dataset was randomly split into training (75%) and test (25%) data sets. AutoQSAR employs different machine learning methods to build either continuous or categorical QSAR models. In this study, a continuous model for binding affinities was built. The quality of the generated models was assessed by using the ranking score, R-squared (R^2^), Q-squared (Q^2^), and root-mean-squared error (RMSE) values. Additionally, the top 10 compounds screened using MD and MM-GBSA scores were assigned to a prediction set. The binding affinities for these 10 compounds were predicted using the generated AutoQSAR model.

### 2.7. Chemicals

Montelukast (M), telmisartan (T), and dimethyl sulfoxide (DMSO) were purchased from Sigma-Aldrich (St. Louis, MO, USA). Compound stock solutions (20 mM) were prepared in DMSO (freshly prepared or stored as single-use aliquots at −20 °C and used within a week of preparation).

### 2.8. Vero Cell Culture

Vero cells (ATCC-CCL81) were maintained in M199 media supplemented with 10% fetal bovine serum and 2% antibiotic-antimycotic. All cells were maintained at 37 °C and 5% CO_2_ in a humidified incubator.

### 2.9. Cell Viability Assay

Vero cells were plated on 24-well plates and incubated at 37 °C and 5% CO_2_ for 24 h. Prior to the addition of the compounds, media was removed. Dilutions of M and T were prepared in M199 cell culture media with a final DMSO concentration of 0.5%. Dilutions of 1, 10, 25, 50, 75, and 100 µM were added to the Vero cells in triplicate. Cells were observed every 24 h for 72 h to observe a cytopathic effect (CPE). The wells containing only M199 and M199 with 0.5% DMSO were treated as negative controls.

Cell viability of M and T in Vero cells was also tested using the CellTiter 96^®^ Non-Radioactive Cell Proliferation Assay (MTT) kit (G4000, Promega, WI, USA) according to the manufacturer’s instructions.

### 2.10. Antiviral Plaque Assay

Dilutions of M and T were prepared in M199 cell culture media and mixed 1:1 with SARS-CoV-2 (SARS-CoV-2, 2019-nCoV/USA-IL1/2020) and incubated at 37 °C for 1 h. Viral work dilution contained 80 plaque-forming units per well, prepared from 2.1 × 10^6^ PFU/mL viral stock (multiplicity of infection; MOI = 0.00016). A final DMSO concentration of 0.5% was maintained for all mixtures. Following incubation, the mixtures were added to Vero cell 6-well plates in triplicate and incubated for 1 h at 37 °C. After incubation, a 0.5% agarose overlay solution was added to cell monolayers of each well. After 24 h, a secondary 0.5% agarose overlay solution containing neutral red was added to each well. After another 24 h, viral plaques of wells inoculated with dilutions of M and T were counted and compared to the number of plaques observed in the virus-only controls, which were diluted in M199 with 0.5% DMSO. Two virus-free negative controls containing M199 and M199 with 0.5% DMSO. The percent inhibition values were calculated using Equation (3).

(3) $$\mathrm{Inhibition}\;\left(\%\right)=\frac{\mathrm{No}.\;\mathrm{of}\;\mathrm{plaques}\;\mathrm{in}\;\mathrm{virus}-\mathrm{only}\;\mathrm{control}-\mathrm{No}.\;\mathrm{of}\;\mathrm{plaques}\;\mathrm{in}\;\mathrm{test}}{\mathrm{No}.\;\mathrm{of}\;\mathrm{plaques}\;\mathrm{in}\;\mathrm{virus}-\mathrm{only}\;\mathrm{control}}\;\times \;100$$

### 2.11. Antiviral Testing Using RT-qPCR

SARS-CoV-2 Omicron variant (BEI resources; NR-56520) was propagated in Vero E6-TMPRSS2-T2A-ACE2 cells (BEI resources; NR-54970) in the BSL3 laboratory facility. For antiviral testing, cells in DMEM media supplemented with 10% FBS and 1% antibiotic were infected with virus (MOI 0.15) and incubated at 37 °C, 5% CO_2_ for 1 h. Next, medium was aspirated, cells were washed with 300 μL DPBS and treated with the drug in 300 μL media followed by incubation at 37 °C, 5% CO_2_. At 48 h, 50 µL of cell supernatant was collected in 50 µL lysis buffer (1:1), boiled at 95 °C for 15 min, and performed RT-qPCR using 7 µL of 200× diluted sample as per protocol established earlier [40]. Viral genome copies were quantified against a standard prepared from heat-inactivated SARS-CoV-2 (BEI resource, NR-52286) and compared for drug effect. After sample collection, cells were fixed in 10% formalin and stained with 1% crystal violet to assess the presence or absence of CPE.

### 2.12. Enzyme-Linked Immunosorbent Assay (ELISA)

The ability of M and T to inhibit the interaction of spike RBD-ACE2 proteins was evaluated by using the SARS-CoV-2 Inhibitor Screening Kit (AG-48B-0001-KI01, AdipoGen Life Sciences, CA, USA). The recombinant Fc-tagged Spike RBD protein (50 ng/well) was coated onto a 96-well ELISA microplate. After overnight incubation (~16 h) at 4 °C, the wells were blocked using Blocking Buffer and incubated at room temperature for 1 h. Different drug concentrations (100, 50, and 25 µM) were added and wells were incubated for 1 h at room temperature. The DMSO concentration was maintained constant (0.1%) for each drug concentration tested (as per the manufacturer’s instructions). As a positive control (PC), hACE2 mAb AC384 (included in the kit) diluted in 1X ELISA buffer (15 nM) was added to the PC wells. The reaction was initiated by adding hACE2 biotin-labeled protein (50 ng/well) diluted in 1X ELISA Buffer to each well and incubated at 37 °C for 1 h. Streptavidin-HRP (dilution 1:1000 in 1X ELISA Buffer) was added to each well and incubated at room temperature for 1 h. The chromogenic reaction was initiated by adding TMB K-Blue Aqueous to each well and incubating at room temperature for 5 min. The reaction was terminated by adding the Stop Solution (2 M H_2_SO_4_), and absorbance at 450 nm was measured using Synergy H1 Hybrid Multi-Mode Microplate Reader (BioTek Instruments, Winooski, VT, USA). The washing procedure (3 × 100 µL 1X Wash Buffer) was performed after coating, blocking, and addition of ACE2 and HRP steps. The wells without inhibitor solution contained only hACE2 protein and were considered 100% activity wells compared to the test and PC wells.

(4) $$\mathrm{Activity}\;\mathrm{Remaining}\;(\%)=\frac{{\mathrm{OD}}_{450\;\mathrm{nm}}\;\mathrm{of}\;\mathrm{test}\;\mathrm{well}}{{\mathrm{OD}}_{450\;\mathrm{nm}}\;\mathrm{of}\;\mathrm{no}\;\mathrm{inhibitor}\;\mathrm{well}}\;\times \;100$$

### 2.13. Biolayer Interferometry (BLI)

The binding kinetics of M and T analytes on SARS-CoV-2 RBD protein were studied using an Octet^®^ R4 system (Sartorius Corporation, Bohemia, NY, USA). Experiments were conducted at 30 °C and a buffer system consisting of 10 mM phosphate buffer, pH 7.4, 137 mM NaCl, 2.7 mM KCl, 0.01% BSA, 0.002% Tween 20, and 0.5% anhydrous dimethyl sulfoxide (DMSO) was used. Recombinant his-tagged SARS-CoV-2 RBD protein wild-type (40592-V08H, Sino Biological US Inc., Wayne, PA, USA) and RBD Omicron variant (40592-V08H129, Sino Biological US Inc., Wayne, PA, USA) at a concentration of 5 μg/mL was loaded on HIS1K biosensors (Sartorius Corporation, Bohemia, NY, USA) for 300 s, followed by a baseline step. The association and dissociation profiles of the analytes were measured at various concentrations. A reference sample was used for baseline correction using a biosensor loaded in the same manner with 0 μM M and T. Reference biosensors were used for each concentration of the analyte without RBD loading. The final binding curves were analyzed with the Octet Analysis Studio Software (Sartorius Corporation, Bohemia, NY, USA) using the 1:1 global-fitting model. 

For the analysis of M and T competing with ACE2 for binding to RBD (both WT and the Omicron variant), after loading and baseline step the biosensors were dipped in 5 μg/mL mFc-tagged ACE2 (10108-H05H, Sino Biological US Inc., Wayne, PA, USA) for 300 s, then moved onto 5 μg/mL ACE2 (control sample), 5 μg/mL ACE2 and 100 µM M, and 5 μg/mL ACE2 and 100 µM T for another 300 s. Reference biosensors loaded with only 100 µM M or T were used to deduct non-specific binding of respective molecules. A reference sample with only RBD protein loaded was used for baseline correction.

## 3. Results

### 3.1. Molecular Docking

**Figure 2:**
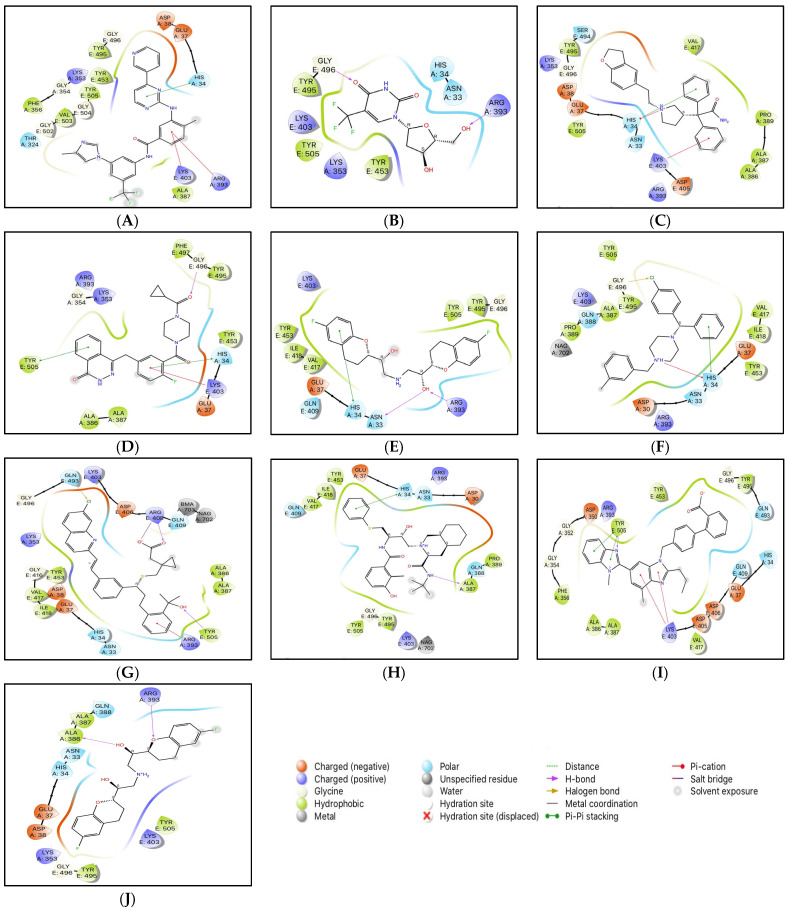
2D interactions of docked protein-drug complexes; (**A**) Nilotinib, (**B**) Viroptic, (**C**) Darifenacin, (**D**) Olaparib, (**E**) Nebivolol, (**F**) Meclizine, (**G**) Montelukast, (**H**) Nelfinavir, (**I**) Telmisartan, and (**J**) Lifitegrast.

The conformations with the highest docking scores for all ten drug molecules with the RBD-ACE2 complex were analyzed for intermolecular interactions that were involved in the formation of stable protein-drug complexes. Table 1 lists the important interacting residues for all docked complexes and Figure 2 represents a schematic representation of the interactions. As indicated in Table 1, Tyr453, Gln493, Ser494, Gly496, Gly502, and Tyr505 are some of the important SARS-CoV-2 RBD residues that directly bind with ACE2. While His34, Glu37, Asp38, Lys353, and Gly354 are some key residues from ACE2 that are involved in RBD binding. At the SARS-CoV-2 RBD-ACE2 interface, Lys353 from ACE2 has been reported as an important virus-binding hotspot that forms a hydrogen bond with the main chain of RBD and a salt bridge with Asp38 from ACE2 [22,41,42]. Electrostatic interactions with Glu37, Asp38, and Lys353 from ACE2 are common for all ten drugs. Except for nilotinib, darifenacin, and meclizine, all screened molecules showed hydrogen bonding with residues at the interface of RBD and ACE2.

Although nilotinib reported the highest docking score (−7.159 kcal/mol) it did not form any hydrogen bonds and mainly interacted with the residues at the active site through polar, hydrophobic, electrostatic, and glycine interactions. Viroptic (−6.919 kcal/mol) and olaparib (−6.41 kcal/mol) interact with Gly496 while montelukast (−6.144 kcal/mol) and telmisartan (−5.961 kcal/mol) interact with Tyr505 from RBD through strong hydrogen bonding. Moreover, all drug molecules showed polar and hydrophobic interactions with His34 from ACE2 and Tyr505 from RBD, respectively. Two drugs, montelukast and meclizine (−6.335 kcal/mol) showed the formation of halogen bonds with Gln493 and Gly496 from RBD, respectively, involving chlorine from the ligand structures as the electron acceptor species. Based on the number and type of interactions formed with the interface residues, the selected drug molecules can be arranged in descending order; montelukast, telmisartan, nilotinib, darifenacin, meclizine, nelfinavir, olaparib, lifitegrast, nebivolol, and viroptic as potential inhibitors of the RBD-ACE2 complex.

### 3.2. MD Simulations

**Figure 3:**
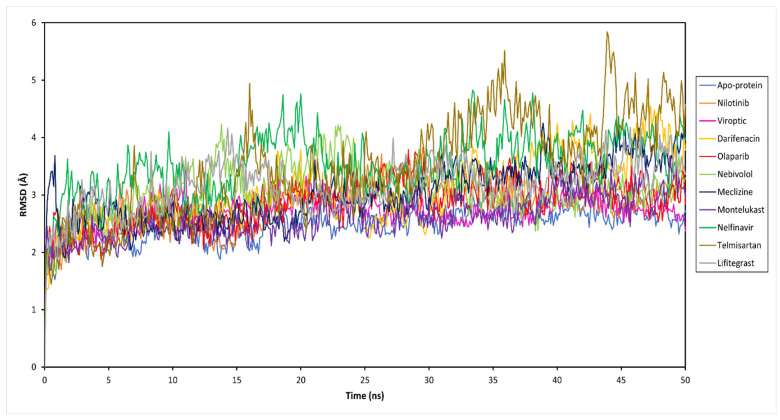
Root mean square deviation (RMSD) of the Cα atoms of the SARS-CoV-2 RBD-ACE2 complex (apo-protein) and inhibitor-bound complexes, plotted as a function of time.

To decipher the structural and conformational changes upon drug binding, all-atom molecular dynamics simulations of 50 ns were performed for all ten complexed systems along with the apo-protein (only RBD-ACE2 complex). The stability of the docked protein-drug complexes was evaluated by measuring the RMSD as a function of simulation time and residual RMSF analysis for the simulation trajectory. This was done by extracting 500 trajectory snapshots for each system at an interval of 100 ps throughout 50 ns. The RMSD analysis revealed that the free and drug-bound RBD-ACE2 systems reached stability after 25 ns with similar RMSD values that oscillated between 2.67 and 4.10 Å (Figure 3 and Table S1). Viroptic and montelukast systems showed Cα deviations similar to the apo-protein indicating the formation of a stable complex compared to nelfinavir and telmisartan systems that showed slightly higher deviations. 

The ligand RMSD indicates the stability of the ligand with respect to the protein and its binding pocket. The ligand RMSD values for the selected drug molecules ranged between 2.77 and 5.86 Å (Table S1). Viroptic reported the lowest deviation, indicating higher stability at the active site compared to nelfinavir and lifitegrast which showed higher deviations. Interestingly, although the binding of telmisartan caused higher structural deviations in the alpha carbons of the protein, lower ligand RMSD indicates that the drug seems to be stable in its binding pocket.

Further, the protein and ligand RMSF values, and the flexibility of each amino acid along the protein chain or ligand atom position, were evaluated throughout the 50 ns simulation. The trajectory of the RBD-ACE2 protein complex alone fluctuated around 1.37 Å. The drug-bound complexes showed Cα fluctuations between 1.29 and 1.69 Å. The binding of drug molecules to the protein exhibited higher fluctuations compared to the apo-protein, except montelukast which reduced the protein conformational flexibility probably due to the higher number of protein contacts. Major fluctuations were typically observed at the tails (N- and C-terminal) in all trajectories.

**Figure 4:**
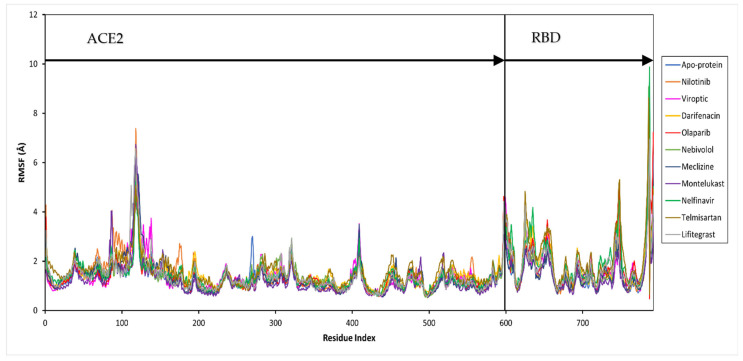
Per residue, root mean square fluctuation (RMSF) of the SARS-CoV-2 RBD-ACE2 complex (apo-protein) and inhibitor-bound complexes. The residues belonging to ACE2 and RBD are shown.

The secondary structure information of the protein is shown in Figure S1. The changes in the ligand atom positions with respect to the protein were less than ~3 kcal/mol for all drug complexes. The overall residual and ligand fluctuations of the RBD-ACE2 apo-protein and drug complexes are summarized in Table S1, and shown in Figure 4.

**Figure 5:**
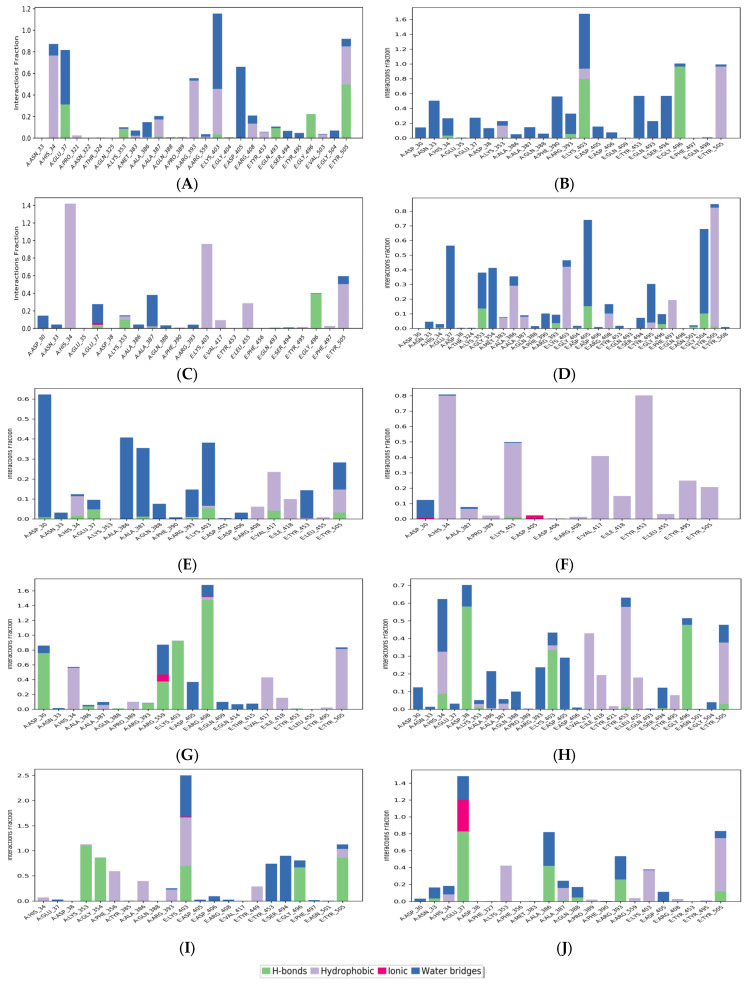
Protein-ligand contacts for (**A**) Nilotinib, (**B**) Viroptic, (**C**) Darifenacin, (**D**) Olaparib, (**E**) Nebivolol, (**F**) Meclizine, (**G**) Montelukast, (**H**) Nelfinavir, (**I**) Telmisartan, and (**J**) Lifitegrast, with SARS-CoV-2 RBD-ACE2 complex extracted from the trajectory of 50 ns simulations. The ligand-interacting protein residues are shown along with the fraction of simulation for every type of contact.

Figure 5 shows the histogram of interactions for the protein-drug complexes indicating the type of interactions per fraction of the simulation trajectory. Hydrogen bonding and hydrophobic contacts were the major types of interactions reported by all drug molecules. The common residues involved in hydrogen bonding with the drug complexes were Glu37 and Lys353 from ACE2, and Lys403, Gly496, and Tyr505 from RBD. viroptic, montelukast, nelfinavir, telmisartan, and lifitegrast showed significant hydrogen bond formation with important residues at the interface of RBD and ACE2 proteins. Interestingly, the drug molecules that did not show any hydrogen bonds in the docked complexes reported some hydrogen bonding in the MD trajectory. For instance, nilotinib and darifenacin reported hydrogen bonding with some common residues (A: Glu37, Lys353; E: Gly496, Tyr505). While meclizine did not report any significant hydrogen bonding even in the MD simulation and mainly interacted via strong hydrophobic interactions. Significant hydrophobic contacts were observed with His34 from ACE2 and Lys403, Val417, Tyr453, and Tyr505 from RBD. Darifenacin, lifitegrast, montelukast, nilotinib, telmisartan, and viroptic showed strong interactions with some residues that were maintained for more than 70% of the simulation time. Comparatively, olaparib, meclizine, montelukast, nelfinavir, and telmisartan made more contact with the RBD residues while viroptic and lifitegrast interacted more with the ACE2 residues. Exceptions were nilotinib, darifenacin, and nebivolol which interacted equally with both proteins. Overall, viroptic, olaparib, montelukast, and telmisartan showed strong interactions (hydrogen bond or hydrophobic contact) with critical RBD residues that are directly involved in the molecular recognition of the ACE2 receptor, suggesting probable inhibitors of the RBD-ACE2 complex.

### 3.3. PCA

**Figure 6:**
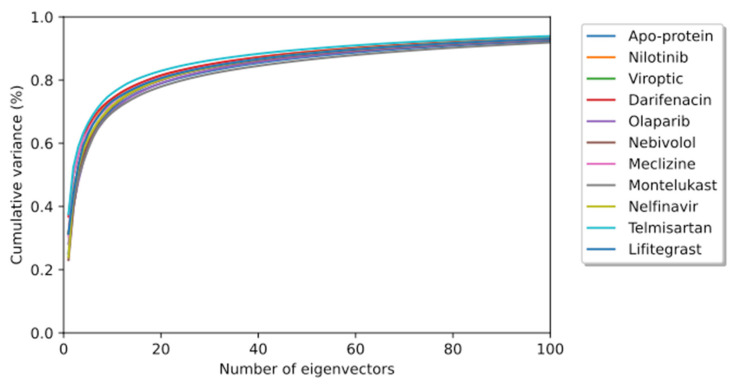
The cumulative contribution of the top 100 eigenvectors to the variance of the overall protein dynamics of the SARS-CoV-2 RBD-ACE2 complex (apo-protein) and protein-drug complexes in a 50 ns simulation trajectory.

**Figure 7:**
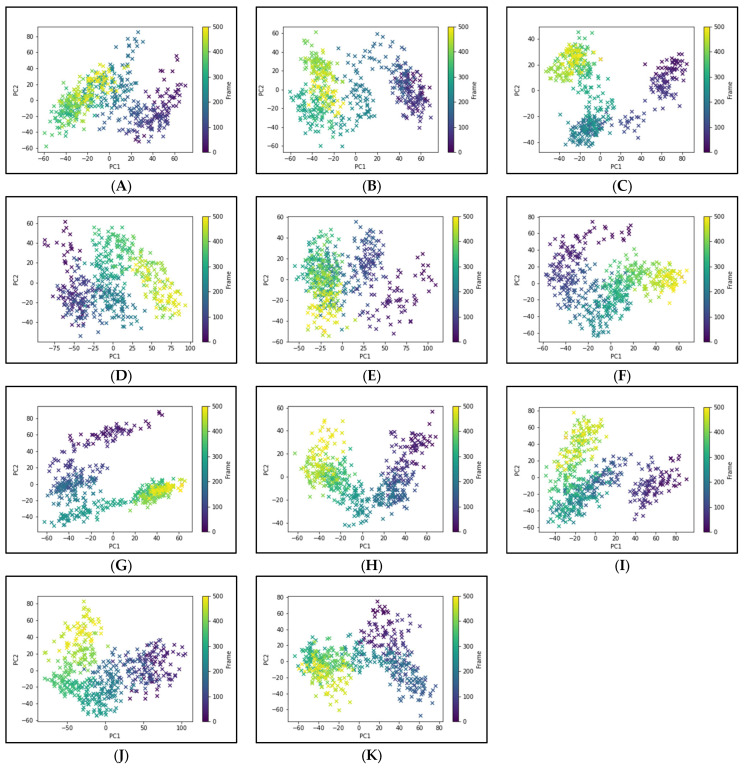
Principal component analysis of 50 ns MD trajectory of free and bound protein. (**A**) SARS-CoV-2 RBD-ACE2 complex (apoprotein), (**B**) Nilotinib, (**C**) Viroptic, (**D**) Darifenacin, (**E**) Olaparib, (**F**) Nebivolol, (**G**) Meclizine, (**H**) Montelukast, (**I**) Nelfinavir, (**J**) Telmisartan, and (**K**) Lifitegrast. The motion of alpha carbon atoms of protein is indicated by a color change from violet to green to yellow for the duration of the simulation trajectory.

To elucidate the essential motion and protein conformational space, MD trajectories of the SARS-CoV-2 RBD-ACE2 complex (apo-protein) and its inhibitor complexes were subjected to essential dynamics analyses/PCA. Although the overall motion of the Cα atoms was distributed over 502 eigenvectors, the top 10 eigenvectors account for more than 70% of the motions, shown in Figure 6. The first two principal components (PC1 and PC2) together captured approximately 40–52% variance in the collective motion of the Cα atoms in the apo-protein and complexes. The projection of the displacement of Cα atoms along the PC1 and PC2 over the MD trajectory of 50 ns simulation for all systems is shown in Figure 7. It can be observed that the scale of PC2 is larger for the apo-protein (Figure 7A) compared to the other drug complexes, indicating that the RBD-ACE2 complex is comparatively more flexible with respect to the motion projected along PC2. Viroptic, darifenacin, olaparib, and telmisartan showed a relatively larger range of PC1 compared to the apo-protein. Also, nebivolol, meclizine, and montelukast occupied a lesser conformational space compared to the apo-protein and other drug complexes, indicating a restricted motion of the Cα atoms. Amongst these montelukast considerably reduced the overall flexibility of the RBD-ACE2 complex. Additionally, the color gradient from violet to yellow in the PCA plots illustrates fluctuations in the distribution of clustered motion during the MD simulation. The positive limits in the PCA plots indicate positively correlated (along the same direction) motion, whereas the negative limits indicate anti-correlated (opposite-direction) motion. The PCA plots in Figure 7 indicate substantially correlated motions along both PC1 and PC2 for the apo-protein and drug complexes.

### 3.4. Binding Energy Calculations

The free energy of binding was calculated for each protein-drug complex to evaluate the affinity of the selected drugs to the binding pocket. The MM-GBSA binding free energies were computed for the last 25 ns of the simulation trajectory. The total ΔG_Bind_ and contributing energetic components along with ligand strain energy are summarized in Table 2. 

The drugs reported binding free energies ranging between −67.69 and 34.51 kcal/mol. The analysis of individual energy components revealed the considerable contribution of ΔG_Lipophilic_ (lipophilic energy) and ΔG_vdW_ (van der Waals energy) to the total free energy of binding for all protein-drug complexes. Additionally, no significant conformational ligand strain energy was observed for all drug molecules. The ligand-binding strain energy characterizes the energy strain experienced by the ligand when adapting to its bioactive conformation from its unbound conformation. Most molecules reported ligand strain energies below 5 kcal/mol while others were still lower than the energy range reported for small molecules binding to protein (0 to ~25 kcal/mol) [43]. Telmisartan reported the highest affinity (−67.69 ± 5.51 kcal/mol) amongst all drug molecules and has a lower ligand strain which indicates its high potential binding at the active site. Although montelukast has a considerably high affinity its ligand strain energy is the maximum among all drug molecules. The top two drugs, montelukast (M) and telmisartan (T), with the best (most negative) MM-GBSA scores, were selected for experimental validation. 

The effect of M and T was also investigated for the RBD-ACE2 complex for the Omicron variant, a SARS-CoV-2 VOC, using MD simulations. Both M and T recorded lower MM-GBSA scores compared to those for the wild-type RBD-ACE2 complex (Table S2). 

### 3.5. AutoQSAR Model

**Figure 8:**
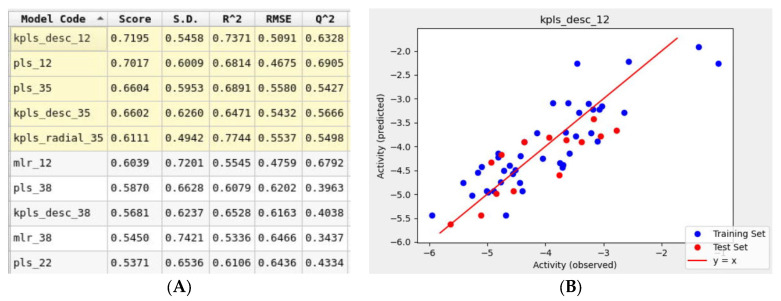
(**A**) Snapshot of report and performance parameters for top ten AutoQSAR models. (**B**) Correlation plot of Glide docking binding affinities and predicted binding affinities for the top-ranked kpls_desc_12 AutoQSAR model.

To support the in silico results obtained using molecular mechanics tools, a simultaneous machine learning approach was employed to generate a binding affinity-based QSAR model. An initial learning set was developed using molecular structures of 60 FDA-approved drugs that were used for the Glide docking studies. This learning set was randomly split into training and test sets with a default ratio of 75/25%. The ten drugs listed in Table 1. were not included in the learning set and were assigned to a prediction set. The initial iteration of the top-ranked AutoQSAR model had low R^2^ and Q^2^ values Figure S2. This model was generated using the kernel-based partial least squares regression (KPLS) method that uses physicochemical and topology-based descriptors. Three outliers that were identified had a common feature: a high number of hetero atoms (>6). Once removed from the learning set, the generated models yielded higher performance parameters. The top-ranked ten QSAR models shown in Figure 8, ranked in decreasing order of model quality scores, were generated using various machine-learning methods. Six of the ten models reported a Q^2^ greater than 0.5, which is considered an acceptable threshold for satisfactory performance [44]. The KPLS models were identified as a majority of the top-five ranked models. The best-performing model (kpls_desc_12) was generated by the descriptor-based KPLS method, using the 12th spilt of the learning set. This model had a quality score of 0.7195, R^2^ of 0.7371, RMSE of 0.5091, and Q^2^ of 0.6328. This model was used to predict the binding affinities (Table 3) of the drugs assigned to the prediction set. The model underestimated the binding affinities by 2 kcal/mol for most drugs including montelukast and telmisartan.

### 3.6. In Vitro Anti-SARS-CoV-2 Activity

**Figure 9:**
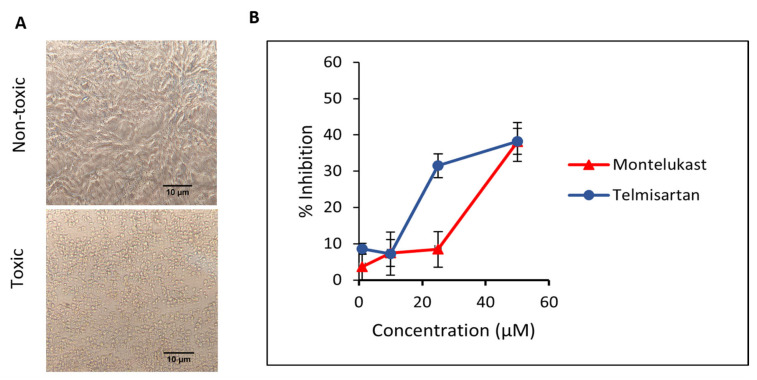
(**A**) The representative images for the cell viability assay depicting toxic and non-toxic effects on Vero cells were taken after 48 h (duration of the antiviral assay) at 20× magnification captured using. (**B**) Inhibition of SARS-CoV-2 in Vero cells when incubated with montelukast and telmisartan. Results are shown as mean ± SEM (n = 3).

First, the cytotoxic effect of M and T was evaluated. Different dilutions of compounds were incubated on Vero cells and cells were observed for morphological changes at 24, 48, and 72 h. For M, toxicity was visible at 100 µM concentration and slight cell clearance at 75 µM after 24 h. On the contrary, cells were healthy at 24 h for all concentrations of T and showed early signs of cell stress at 48 h for 100 and 75 µM. Minimal toxicity or cell stress was observed for concentrations of M and T below 50 µM for up to 48 h (duration of the antiviral plaque assay), hence those concentration ranges were tested against the virus. The representative data for the cell viability assay (Figure 9A) depicting toxic and non-toxic effects on Vero cells are shown in the images taken at 20× magnification after 48 h. The cell viability for M and T in Vero cells using MTT assay is shown in Figure S3. This data is consistent with previously published cell viability data in Vero cells for M [19] and T [45].

Next, we evaluated the ability of M and T to inhibit SARS-CoV-2 WT replication in a plaque reduction assay. To study the effect on entry, different dilutions of M and T were first incubated with the virus at 37 °C for 1 h and then added to Vero cells. At 50 µM concentration, both M and T were able to suppress ~38% of plaque formation. As shown in Figure 9B, a dose-dependent response is visible for the tested concentrations of M and T.

**Figure 10:**
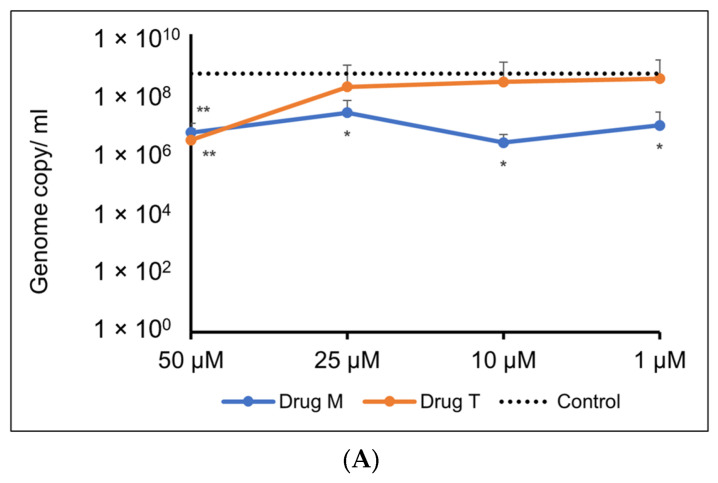

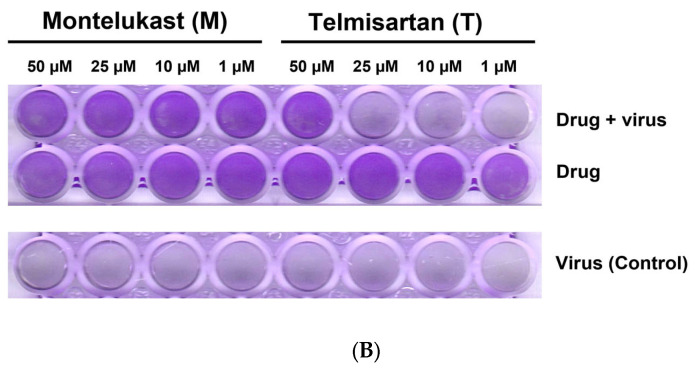
(**A**). Representative line graph showing inhibition of SARS-CoV-2 genome replication in Vero E6-TMPRSS2-T2A-ACE2 cells when incubated with montelukast or telmisartan. Vero E6-TMPRSS2-T2A-ACE2 cells were infected with the SARS-CoV-2 Omicron variant (MOI = 0.15) in the presence or absence of the drug. RT-qPCR was carried out after 48 h to quantify genome copy. Sample means were compared to the control mean using student’s *t*-test. * *p* < 0.05, ** *p* < 0.01 (N = 11 to 24 data points). (**B**) Representative image of Vero E6-TMPRSS2-T2A-ACE2 cells stained with 1% crystal violet to observe cytopathic effect subsequent to SARS-CoV-2 Omicron variant infection and drug treatment.

For the Omicron variant, M showed an inhibitory response of virus replication in all the doses tested, but telmisartan was effective only at 50 µM (Figure 10A,B). 

### 3.7. ELISA

**Figure 11:**
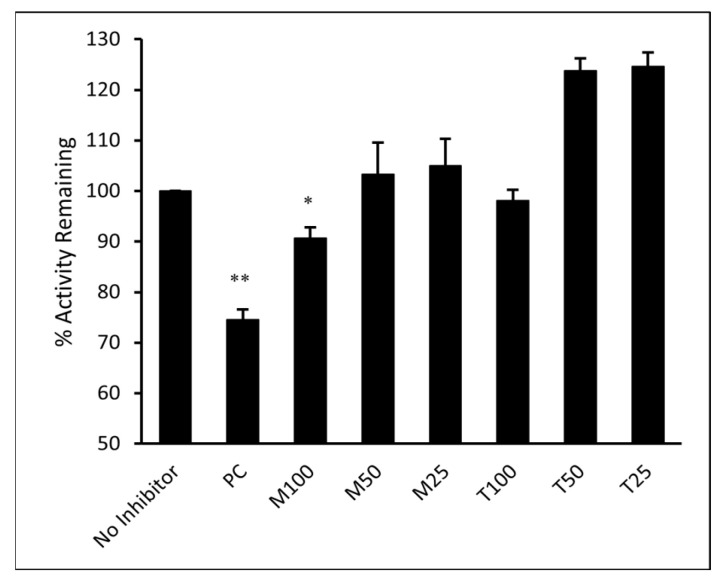
Montelukast (M) and telmisartan (T) were tested at various concentrations (100, 50, and 25 µM) to evaluate their ability to inhibit the binding of ACE2 onto immobilized SARS-CoV-2 spike RBD in vitro. hACE2 mAb AC384 (15 nM) was used as a positive control (PC). Results are shown as mean ± SEM (n = 2). ** *p* < 0.01; * *p* < 0.05 vs no inhibitor well.

The ability of M and T to directly inhibit the binding of SARS-CoV-2 spike RBD to its cellular receptor ACE2 was evaluated in vitro. The inhibitory activity of M and T was tested at 25, 50, and 100 µM concentrations. As shown in Figure 11, the results were compared to the sample that contained only ACE2 protein and no inhibitor (assumed 100% activity). Montelukast significantly (*p*-value < 0.05) reduced spike RBD activity by 11% at 100 µM concentration. However, T did not show any significant effect on enzyme activity even at the highest concentration tested. Monoclonal antibody (mAb) AC384, specifically binds to human ACE2 (hACE2) protein, was used as a positive control (PC). It showed 26% loss of enzyme activity at 15 nM concentration (*p*-value < 0.01).

### 3.8. BLI

**Figure 12:**
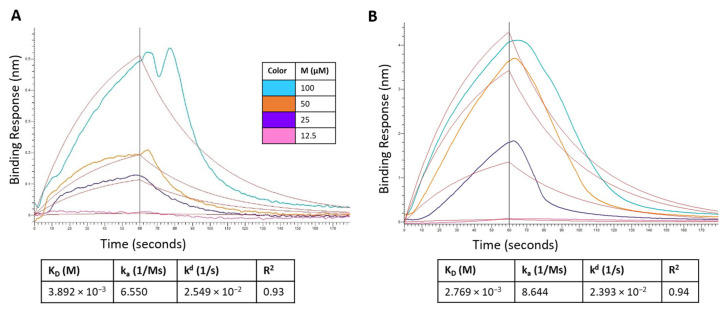

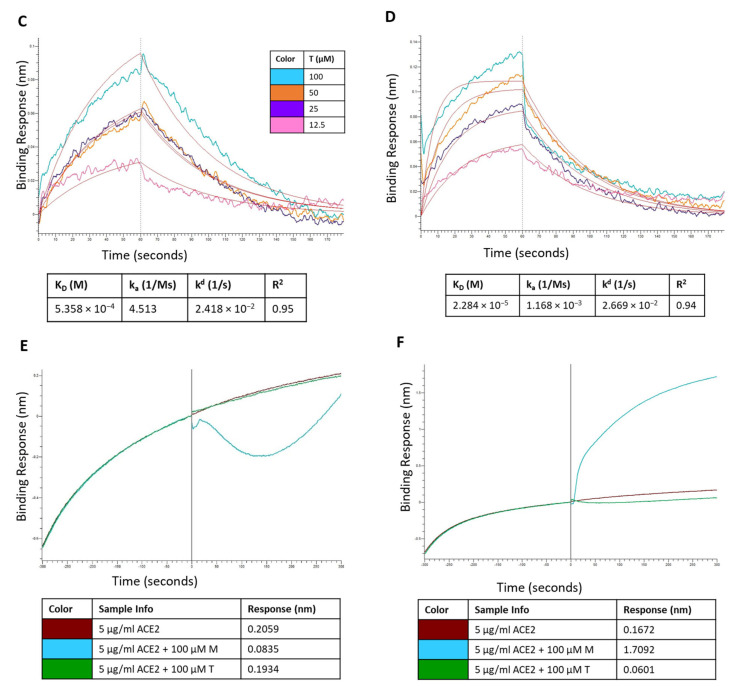
Sensorgram for the binding of montelukast at different concentrations onto immobilized: (**A**) RBD-WT and (**B**) RBD-Omicron variant. Sensorgram for the binding of telmisartan at different concentrations onto immobilized (**C**) RBD-WT and (**D**) RBD-Omicron variant. Competition analysis of montelukast and telmisartan competing with ACE2 for binding onto immobilized: (**E**) RBD-WT and (**F**) RBD-Omicron variant.

The binding kinetics of montelukast and telmisartan to the RBD of SARS-CoV-2 spike protein (WT and Omicron) were evaluated using BLI. The analysis showed (Figure 12A) that M binds to the SARS-CoV-2 RBD-WT protein with an association rate (k_a_) of 6.550 M^−1^ s^−1^ and dissociation rate (k_d_) of 2.549 × 10^−2^ s^−1^. This resulted in an equilibrium affinity constant (K_D_) of 3.892 mM which is calculated as a ratio of the k_d_ and k_a_ values. The affinity value indicates that 50% of the RBDs on the surface spike glycoproteins will be occupied at millimolar concentrations of montelukast. Montelukast showed a similar affinity to the RBD of Omicron variant but had a higher response value (~10 times of recorded response for RBD-WT at 100 µM) as shown in Figure 12B. This suggested that montelukast has a potential secondary binding site on RBD-Omicron. Telmisartan recorded an affinity of 0.5358 mM (Figure 12C) and 22.8 µM (Figure 12D) to RBD-WT and RBD-Omicron, respectively. 

Additionally, a competition assay was performed to examine if montelukast would compete with ACE2 to bind with RBD at the interface of the two proteins. The biosensors with immobilized RBD were dipped in ACE2 solution and then dipped in samples containing only ACE2 (control), and mixture of ACE2 with either M or T. For RBD-WT, as shown in Figure 12E, the control sample recorded a response of 0.2059 nm. The response observed for sample containing 100 µM T was 0.1934 which was close to the response of control sample. However, in the presence of 100 µM M the response recorded was 0.0835 nm (~60% reduction in response). These results provide evidence that M binds to the RBD-WT protein at the interface of ACE2 protein binding.

For RBD-Omicron, M recorded a higher response compared to control. This is consistent with the above binding results. Hence, the competition assay for M binding to RBD-Omicron in the presence of ACE2 was inconclusive likely due to the presence of potential secondary binding site. On the contrary, telmisartan competitively bound to RBD at the ACE2 binding interface and reduced the response by ~64% compared to the control. 

## 4. Discussion

Emerging viral infections that cross the species barrier are difficult to contain, complicating the development of target-specific drugs and vaccines. Although the ongoing COVID-19 pandemic has a low mortality rate, it is highly infectious compared to the previous coronavirus outbreaks [46]. Although experts believe that eradicating SARS-CoV-2 would be a difficult task given its high transmission rate and broad-spectrum clinical manifestations, it is imperative to identify potent drugs that can help reduce disease and potentially contain the spread of the virus. In such cases, drug repurposing is a practical approach rather than an expensive and time-consuming process of developing a new drug. In the past, computational approaches have proven beneficial for repositioning existing drugs against novel protein targets [47]. Our efforts in the present study, through the application of molecular mechanics and machine learning tools, were directed toward screening clinically approved drug molecules as potential inhibitors of the SARS-CoV-2 RBD-ACE2 complex.

Molecular docking revealed ten FDA-approved drugs as potential inhibitors of the SARS-CoV-2 RBD-ACE2 complex. These include nilotinib and olaparib (anticancer), viroptic and nelfinavir (antivirals), darifenacin (bladder relaxant), nebivolol and telmisartan (anti-hypertensive), meclizine (antihistamine), montelukast (anti-inflammatory), and lifitegrast (ophthalmic solution for treating dry eye syndrome) [48]. Amongst these, nilotinib, olaparib, nebivolol, telmisartan, montelukast, and nelfinavir have been tested in-vitro against SARS-CoV-2 [19,21,49,50,51,52,53,54]. A case study of COVID-19-induced vestibular neuritis reported the use of meclizine for symptomatic treatment [55]. Another study reported mitigated apoptosis in human pluripotent stem cell-derived cardiomyocytes due to a potential role of meclizine in maintaining ATP balance, thus suggesting its use for COVID treatment [56]. Two retrospective assessments of COVID-19-positive patients revealed the potential of montelukast as an effective treatment against SARS-CoV-2 [57,58]. 

Our computational results provide crucial structural and mechanistic insights into the binding of these drug molecules at the ACE2 molecular recognition site of RBD. Further intermolecular interaction analyses revealed that all screened drugs showed significant interactions via hydrogen bonding and hydrophobic contacts with critical residues at the interface of RBD and ACE2 proteins. The most common residues were His34, Glu37, and Lys353 from the ACE2 protein and Lys403, Val417, Tyr453, Gln493, Gly496, and Tyr505 from RBD. MM-GBSA binding free energy analysis revealed the affinity of drug molecules in the decreasing order: telmisartan, montelukast, nelfinavir, darifenacin, nilotinib, lifitegrast, meclizine, nebivolol, olaparib, and viroptic. Although telmisartan is an angiotensin II receptor blocker, it showed significant interactions with the RBD residues as well. The post-MD analysis is consistent with this phenomenon that telmisartan forms strong hydrogen bonds with Lys353 and Gly354 of ACE2 compared to Gly496 and Tyr505 of RBD. Essential dynamics analysis of the alpha carbon atoms of the protein in the telmisartan complexed system indicated increased flexibility along PC1 compared to the apo-protein. The dynamics of the protein-montelukast system indicated the formation of a relatively stable complex. While montelukast reported a slightly lower affinity, our PCA indicated that it significantly restricted the motion of the Cα atoms along both PC1 and PC2 when compared to the other protein-drug complexes. 

In our antiviral assay, both montelukast and telmisartan at 50 µM showed 38% inhibition of SARS-CoV-2 in Vero cells. Our results are within the range of values published in previous studies that assessed the in vitro activity of montelukast against SARS-CoV-2 in VeroE6 cells [19,52] and activity of telmisartan against SARS-CoV-2 in Caco-2 cells [21]. In the genome quantitation assay for the Omicron variant, M showed an inhibitory response of virus replication in all the doses tested, but telmisartan was effective only at 50 µM. Identifying the mechanism of action (MOA) and pinpointing the protein target is an important aspect of drug development. At 100 µM concentration montelukast showed ~60% reduction in ACE2 binding response to RBD-WT for the BLI competition assay and ~11% inhibition in ELISA. It must be noted that BLI reports real-time binding response whereas ELISA is an end-point detection method, which explains the difference in binding inhibition. Comparing biochemical and antiviral assay results suggests that montelukast might have a synergistic inhibitory effect on SARS-CoV-2. The data is consistent with a previous study that evaluated the inhibitory effect of montelukast on the 3C-like protease enzyme of SARS-CoV-2 [19]. This may also be plausible for the Omicron variant since a study found that the mutation on the 3CL-like protease enzyme of Omicron SARS-CoV-2 decreases thermal stability without compromising catalysis or small-molecule drug inhibition [59]. Similarly, although telmisartan did not significantly affect the binding of RBD to ACE2 in our enzyme binding inhibition assay, our antiviral results suggest the use of telmisartan against SARS-CoV-2 is likely due to its role as a cellular target antagonist, as previously described [60].

Studies have reported that patients with cardiovascular diseases, diabetes, respiratory system diseases, immune system diseases, and severe obesity are prone to severe illness from the virus [61]. Telmisartan has been suggested for treatment in these patients to control high blood pressure [62,63]. Montelukast is an anti-inflammatory agent effective in the treatment of asthma [64] and has shown antiviral activity against the influenza A virus, zika virus, hepatitis C virus, and respiratory syncytial virus [65,66,67,68,69]. Since montelukast and telmisartan are clinically approved drugs their pharmacodynamics and pharmacokinetics have been well-studied for over two decades. Studies have indicated that high doses of montelukast (10–200 mg/day) and telmisartan (20–160 mg/day) are well tolerated in patients with no significant clinical manifestations [70,71]. However, it must be noted that the FDA recently added a warning regarding the use of montelukast due to its proposed association with neuropsychiatric events [72].

## 5. Conclusions

The current study demonstrates the ability of a comprehensive computational approach, including structure-based molecular docking, all-atomistic MD simulations, and MM-GBSA binding energy estimation, for the identification of viable repurposable drugs against SARS-CoV-2 spike RBD. Ten FDA-approved drugs were identified as potential SARS-CoV-2 inhibitors, of which the efficacy of two drugs, montelukast, and telmisartan, were tested in vitro. A viral inhibition assay demonstrated these drugs showed 38% inhibition of WT SARS-CoV-2 plaque formation at a non-cytotoxic concentration, suggesting their role in blocking viral entry. Montelukast at 100 µM showed a significant reduction in RBD activity and competed with ACE2 for binding to WT RBD. A genome quantitation assay with the Omicron variant confirmed M to be active at all concentrations, i.e., 50, 25, 10, and 1 µM, while T was only active at 50 µM. The non inhibitory dose tested for M drug was 0.781 µM (Figure S4). BLI studies confirmed that both M and T bind to both WT and Omicron RBD. However, a competition assay of the two drugs in the presence of ACE2 indicated that M competed with ACE2 for binding onto WT RBD; however, the case was not entirely clear with the Omicron variant. T on the other hand competed with ACE2 for the WT, but was not as evident as M; however, T clearly competed with ACE2 for binding onto the RBD of the Omicron variant. Although the observed inhibitory activity of M is low compared to the previously described inhibition via 3C-like protease enzyme, it suggests a potential dual inhibitory effect of montelukast which is plausible for many repurposable drugs.

## Figures and Tables

**Table 1:** List of the ten compounds selected through refined docking score and their important interactions with RBD-ACE2 complex.

Sr. No	Name	XP GScore (kcal/mol)	H-Bond	π-π Stacking	π-Cation	Polar	Hydrophobic	Negative Charged	Positive Charged	Glycine	Halogen Bond
1	Nilotinib	−7.159	-	**A: His34**	A: Arg393; E: Lys403	**A: His34**, Thr324	A: Phe356, Ala387; **E: Tyr453**, Tyr495, Val503, **Tyr505**	**A: Glu37, Asp38**	**A: Lys353**, Arg393; E: Lys403	**A: Gly354**; **E: Gly496**, **Gly502**, Gly504	-
2	Viroptic	−6.919	A: Arg393; **E: Gly496**	-	-	A: Asn33, **His34**	**E: Tyr453**, Tyr495, **Tyr505**	-	**A: Lys353**, Arg393; E: Lys403	**E: Gly496**	-
3	Darifenacin	−6.501	-	**A: His34**	**A: His34**; E: Lys403	A: Asn33, **His34**; **E: Ser494**	A: Ala386, Ala387, Pro389; E: Val417, Tyr495, **Tyr505**	**A: Glu37**, **Asp38**; E: Asp405	**A: Lys353**, Arg393; E: Lys403	**E: Gly496**	-
4	Olaparib	−6.41	**E: Gly496**	**A: His34**	E: Lys403	**A: His34**	A: Ala386, Ala387; **E: Tyr453**, Tyr495, Phe497, **Tyr505**	**A: Glu37**	**A: Lys353**, Arg393; E: Lys403	**A: Gly354**; **E: Gly496**	-
5	Nebivolol	−6.368	A: Asn33, Arg393	**A: His34**	-	A: Asn33, **His34**; E: Gln409	E: Val417, Ile418, **Tyr453**, Tyr495, **Tyr505**	**A: Glu37**	A: Arg393	**E: Gly496**	-
6	Meclizine	−6.335	-	**A: His34**	**A: His34**	A: Asn33, **His34**, Gln388	A: Ala387, Pro389; E: Val417, Ile418, **Tyr453**, Tyr495, **Tyr505**	A: Asp30, **Glu37**	A: Arg393; E: Lys403	**E: Gly496**	**E: Gly496**
7	Montelukast	−6.144	E: Arg408, **Tyr505**	-	A: Arg393	A: Asn33, **His34**; E: Gln409, Gln493	A: Ala386, Ala387; E: Val417, Ile418, **Tyr453**, **Tyr505**	**A: Glu37**, **Asp38**; E: Asp406	**A: Lys353**, Arg393; E: Lys403, Arg408	**E: Gly496**	**E: Gln493**
8	Nelfinavir	−6.063	A: Ala387	**A: His34**	-	A: Asn33, **His34**; E: Gln409	A: Ala387, Pro389; E: Val417, Ile418, **Tyr453**, Tyr495, **Tyr505**	A: Asp30, **Glu37**	A: Arg393; E: Lys403	**E: Gly496**	-
9	Telmisartan	−5.961	**E: Tyr505**	**E: Tyr505**	E: Lys403	**A: His34**; E: Gln409, **Gln493**	A: Phe356, Ala386, Ala387; E: Val417, **Tyr453**, Tyr495, **Tyr505**	**A: Glu37**, Asp350; E: Asp405, Asp406	A: Arg393; E: Lys403	A: Gly352, **Gly354**; **E: Gly496**	-
10	Lifitegrast	−5.701	A: Ala386, Arg393	-	-	A: Asn33, **His34**, Gln388	A: Ala386, Ala387; E: Tyr495, **Tyr505**	**A: Glu37, Asp38**	**A: Lys353**, Arg393; E: Lys403	**E: Gly496**	-

Note: Residues starting with “A” belong to the ACE2 protein and those with “E” belong to SARS-CoV-2 spike RBD (WT). Contact residues that are directly involved in SARS-CoV-2 RBD-ACE2 binding are highlighted in bold.

**Table 2:** The binding free energy analysis for the protein-drug complexes calculated over the last 25 ns of MD trajectories.

Name	ΔG_Coulomb_	ΔG_Covalent_	ΔG_Lipophilic_	ΔG_vdW_	ΔG_GB solv_	ΔG_Bind_	ΔE_Strain_
Nilotinib	−12.33 ± 4.06	4.33 ± 2.57	−17.07 ± 1.75	−54.51 ± 5.00	33.82 ± 3.42	−50.57 ± 6.12	3.77 ± 1.80
Viroptic	−18.27 ± 3.12	2.66 ± 1.12	−6.52 ± 0.45	−32.27 ± 1.29	21.72 ± 2.11	−34.51 ± 3.24	2.63 ± 0.97
Darifenacin	−20.25 ± 9.51	1.85 ± 0.78	−16.28 ± 5.43	−44.29 ± 5.44	29.64 ± 10.96	−52.05 ± 9.26	5.71 ± 5.66
Olaparib	−6.36 ± 3.80	3.28 ± 2.02	−15.91 ± 1.92	−49.06 ± 2.97	27.87 ± 2.99	−43.15 ± 5.12	3.94 ± 1.46
Nebivolol	−22.29 ± 7.77	2.78 ± 0.99	−18.50 ± 1.41	−44.49 ± 2.03	36.69 ± 7.60	−48.48 ± 6.18	2.86 ± 1.37
Meclizine	−29.68 ± 7.63	2.09 ± 0.96	−22.14 ± 1.73	−43.05 ± 2.82	47.27 ± 5.78	−48.57 ± 4.47	2.51 ± 0.84
Montelukast	20.92 ± 13.07	2.92 ± 0.73	−21.38 ± 1.22	−63.13 ± 2.17	6.61 ± 12.47	−61.40 ± 3.85	8.51 ± 2.13
Nelfinavir	−29.11 ± 8.49	1.86 ± 3.19	−17.28 ± 5.00	−49.58 ± 4.65	44.57 ± 6.65	−53.16 ± 12.01	6.78 ± 3.77
Telmisartan	41.34 ± 11.97	4.66 ± 1.88	−20.18 ± 1.03	−65.95 ± 2.44	−20.26 ± 9.41	−67.69 ± 5.51	3.26 ± 0.70
Lifitegrast	−32.99 ± 11.30	1.89 ± 0.99	−16.16 ± 2.38	−39.30 ± 3.24	38.70 ± 9.67	−50.18 ± 7.75	3.22 ± 1.48

All energies are in kcal/mol. Data represented as mean ± standard deviation. ΔG_Coulomb_: Contribution to the binding free energy from the Coulombic energy. ΔG_Covalent_: Contribution to the binding free energy from the covalent energy. ΔG_Lipophilic_: Contribution to the binding free energy from the lipophilic energy. ΔG_vdW_: Contribution to the binding free energy from the van der Waals energy. ΔG_GB solv_: Contribution to the binding free energy from the Generalized Born electrostatic solvation energy. ΔG_Bind_: Total binding free energy. ΔE_Strain_: Ligand strain energy.

**Table 3:** Binding affinities predicted by the kpls_desc_12 model for the prediction set.

Name	Binding Affinity (kcal/mol)	Predicted Affinity (kcal/mol)	RMSE (kcal/mol)
Nilotinib	−7.159	−4.876	2.283
Viroptic	−6.919	−3.892	3.027
Darifenacin	−6.501	−3.445	3.056
Olaparib	−6.41	−4.087	2.323
Nebivolol	−6.368	−5.435	0.933
Meclizine	−6.335	−3.861	2.474
Montelukast	−6.144	−4.266	1.878
Nelfinavir	−6.063	−4.759	1.304
Telmisartan	−5.961	−4.599	1.362
Lifitegrast	−5.701	−5.363	0.338

## Data Availability

The data presented in this study are available on request from the corresponding author.

## Supplementary Materials

The following supporting information can be downloaded at: https://www.mdpi.com/article/10.3390/pharmaceutics15071891/s1, Figure S1: Protein information of SARS-CoV-2 RBD-ACE2 complex (PDB: 6VW1) with secondary structure assessment; Table S1: RMSD and RMSF evaluation for the last 25 ns and entire MD simulation trajectory, respectively; Table S2: Prime/MM-GBSA binding free energy calculations for MD of screened compounds with SARS-CoV-2 RBD-ACE2 complex from wild type (WT), and omicron variant; Figure S2: (A). Snapshot of report and performance parameters for top ten AutoQSAR models from the first iteration of the learning set. (B) Correlation plot of Glide docking binding affinities and predicted binding affinities for the top ranked kpls_desc_46 AutoQSAR model; Figure S3: Cell viability in Vero cells using MTT assay for (A). montelukast and (B). telmisartan; Figure S4: Representative bar chart showing inhibition of SARS-CoV-2 Omicron variant genome replication in Vero E6-TMPRSS2-T2A-ACE2 cells when incubated with montelukast. Data represented as mean ± standard deviation (n = 3 to 4 data points).
