# Supplementary material for: Montelukast and Telmisartan as Inhibitors of SARS-CoV-2 Omicron Variant
Source: Pharmaceutics. 2023 Jul 5;15(7):1891. doi: 10.3390/pharmaceutics15071891 (PMC10385313; doi:10.3390/pharmaceutics15071891)
Supplement: Supplementary file 1 [file pharmaceutics-15-01891-s001.zip › pharmaceutics-2442366-supplementary.pdf]

pharmaceutics

MDPI

Article

Supplementary: Montelukast and Telmisartan as Inhibitors of SARS-CoV-2 Omicron Variant

Nirmitee Mulgaonkar, Haoqi Wang, Junrui Zhang, Christopher M. Roundy, Wendy Tang and Sankar Prasad Chaki, Alex Pauvolid-Corrêa, Gabriel L. Hamer and Sandun Fernando

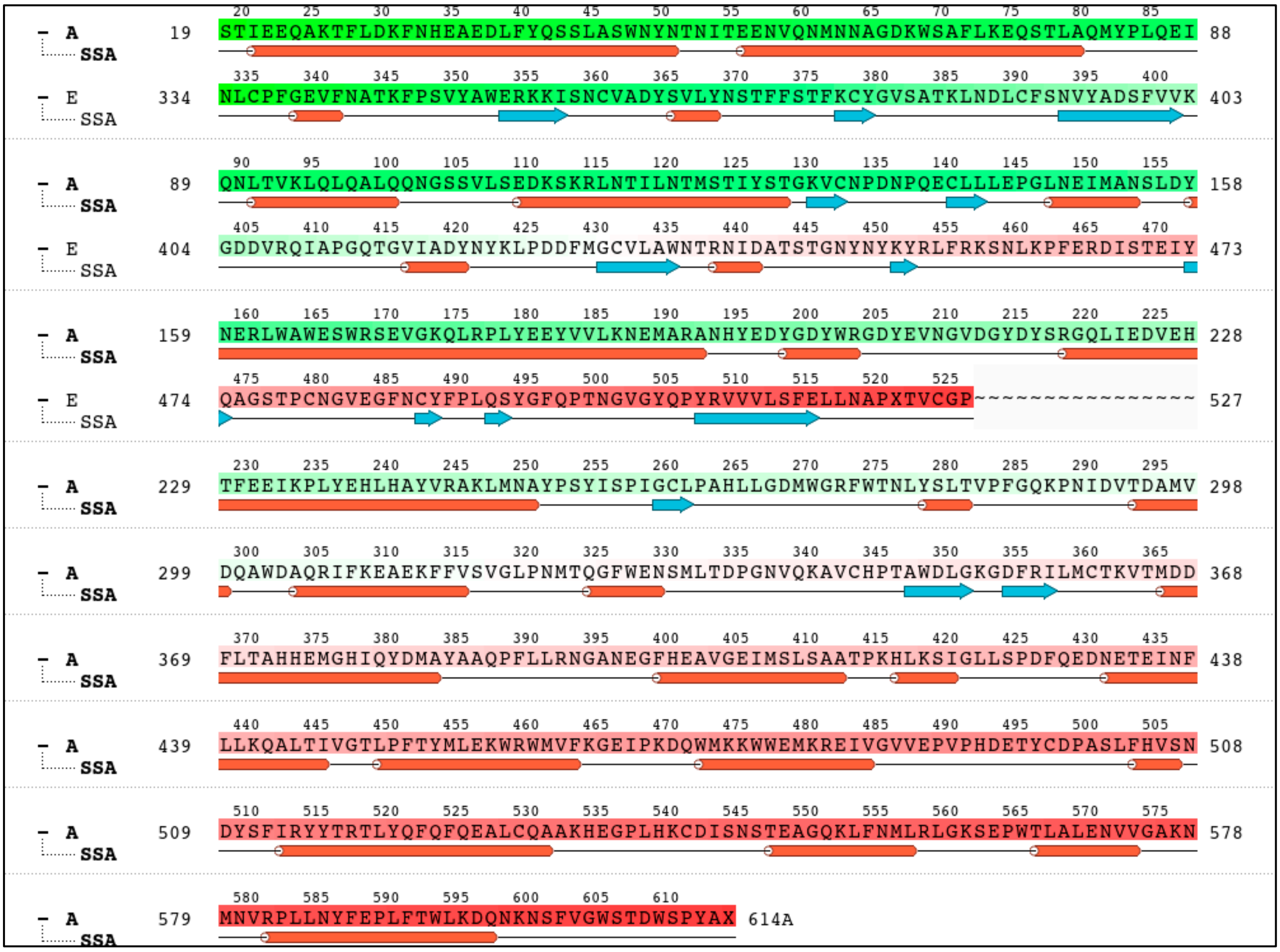

Figure S1. Protein information of SARS-CoV-2 RBD-ACE2 complex (PDB: 6VW1) with secondary structure assessment. Chain E indicates SARS-CoV-2 RBD, and chain A is ACE2 protein. Alpha helix (red cylinders) and beta sheets (blue arrows) are joined by loop regions (black lines).

Table S1. RMSD and RMSF evaluation for the last 25 ns and entire MD simulation trajectory, respectively.

Sr.No.	System	RMSD (Å)		RMSF (Å)	
		Protein Cα	Ligand fit on protein	Protein Cα	Ligand fit on protein
1	Apo-protein	2.67 ± 0.18	-	1.37 ± 0.75	-
2	Nilotinib	3.08 ± 0.25	3.68 ± 0.64	1.46 ± 0.85	1.86 ± 0.78
3	Viroptic	2.74 ± 0.16	2.77 ± 0.21	1.47 ± 0.81	1.33 ± 0.19

4	Darifenacin	3.39 ± 0.56	4.87 ± 0.85	1.58 ± 0.80	1.76 ± 0.32
5	Olaparib	3.13 ± 0.27	4.99 ± 0.63	1.44 ± 0.81	2.26 ± 0.46
6	Nebivolol	3.07 ± 0.24	3.45 ± 0.36	1.42 ± 0.80	1.53 ± 0.27
7	Meclizine	3.40 ± 0.34	4.79 ± 0.44	1.41 ± 0.77	1.90 ± 0.62
8	Montelukast	2.84 ± 0.29	4.59 ± 0.17	1.29 ± 0.69	1.71 ± 0.76
9	Nelfinavir	3.73 ± 0.39	5.86 ± 0.40	1.53 ± 0.74	2.17 ± 0.87
10	Telmisartan	4.10 ± 0.61	3.24 ± 0.26	1.69 ± 0.83	1.39 ± 0.21
11	Lifitegrast	3.38 ± 0.29	5.42 ± 0.61	1.48 ± 0.76	2.89 ± 1.81

Data are represented as mean ± standard deviation

Table S2. Prime/MM-GBSA binding free energy calculations for MD of screened compounds with SARS-CoV-2 RBD-ACE2 complex from wild type (WT), and omicron variant.

Compound	MM-GBSA Binding free energy ΔG_{bind} (kcal/mol)	
	WT	Omicron
Montelukast	-61.40 ± 3.85	-41.16 ± 7.42
Telmisartan	-67.69 ± 5.51	-48.07 ± 7.49

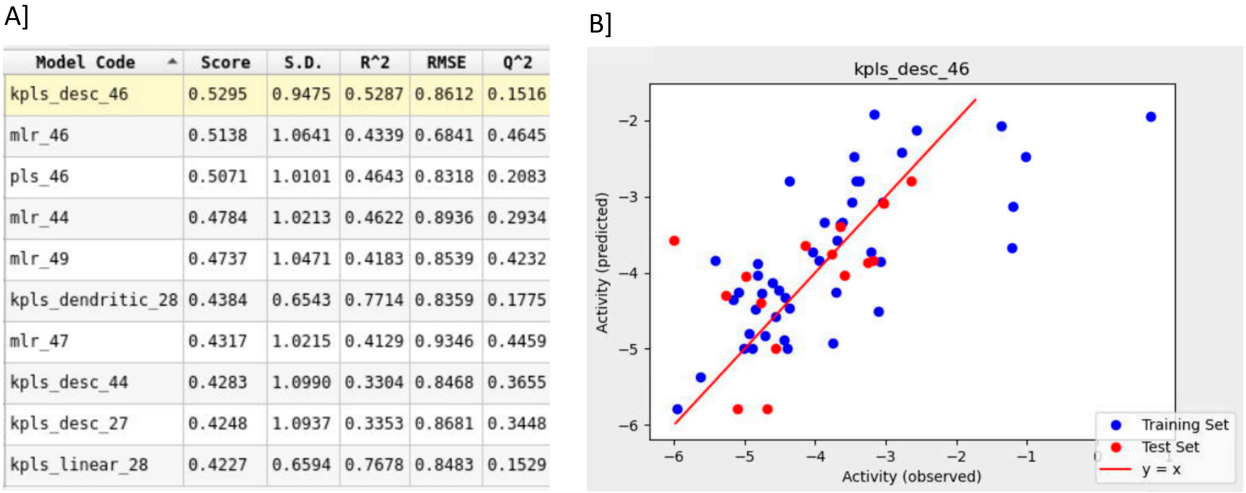

A]

Model Code	Score	S.D.	R^2	RMSE	Q^2
kpls_desc_46	0.5295	0.9475	0.5287	0.8612	0.1516
mlr_46	0.5138	1.0641	0.4339	0.6841	0.4645
pls_46	0.5071	1.0101	0.4643	0.8318	0.2083
mlr_44	0.4784	1.0213	0.4622	0.8936	0.2934
mlr_49	0.4737	1.0471	0.4183	0.8539	0.4232
kpls_dendritic_28	0.4384	0.6543	0.7714	0.8359	0.1775
mlr_47	0.4317	1.0215	0.4129	0.9346	0.4459
kpls_desc_44	0.4283	1.0990	0.3304	0.8468	0.3655
kpls_desc_27	0.4248	1.0937	0.3353	0.8681	0.3448
kpls_linear_28	0.4227	0.6594	0.7678	0.8483	0.1529

Figure S2. A]. Snapshot of report and performance parameters for top ten AutoQSAR models from the first iteration of the learning set. B]. Correlation plot of Glide docking binding affinities and predicted binding affinities for the top ranked kpls_desc_46 AutoQSAR model.

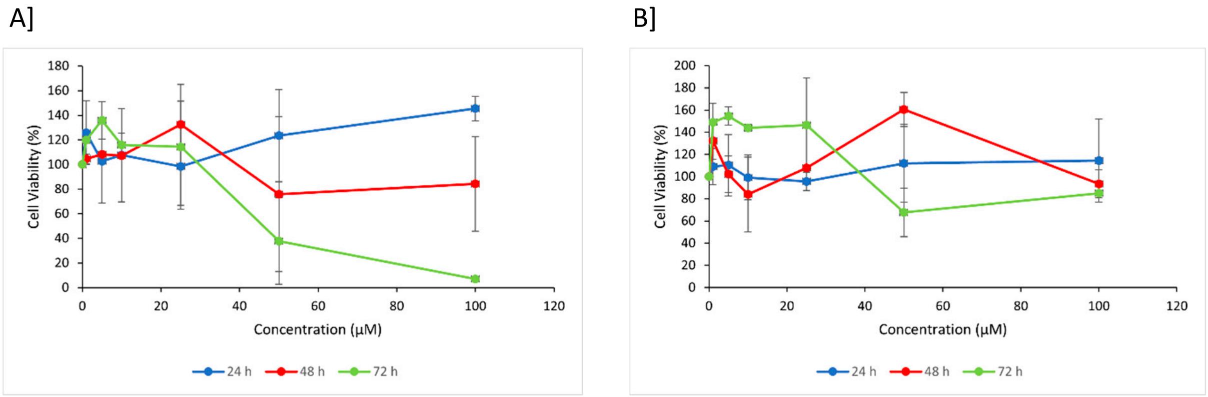

Figure S3. Cell viability in Vero cells using MTT assay for A]. montelukast and B]. telmisartan.

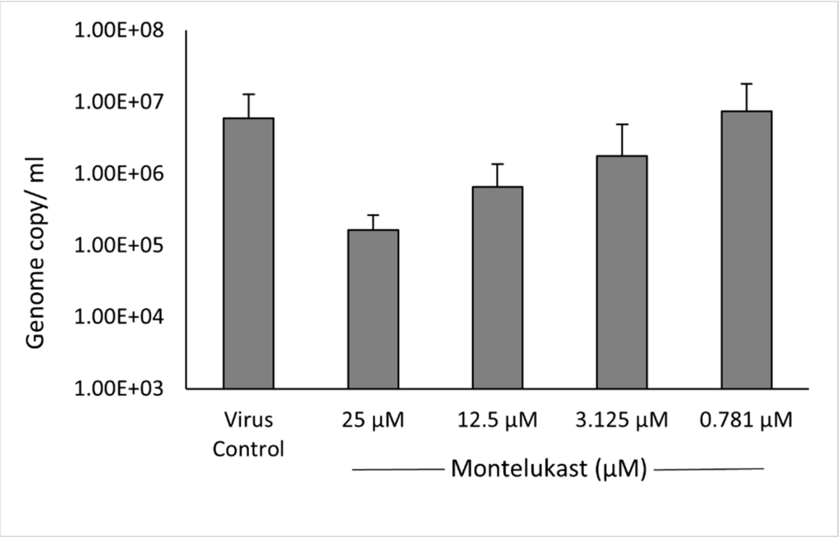

Figure S4. Representative bar chart showing inhibition of SARS-CoV-2 Omicron variant genome replication in Vero E6-TMPRSS2-T2A-ACE2 cells when incubated with montelukast. Data represented as mean ± standard deviation (n = 3 to 4 data points).
